# Endoplasmic reticulum membrane remodeling by targeting reticulon-4 induces pyroptosis to facilitate antitumor immune

**DOI:** 10.1093/procel/pwae049

**Published:** 2024-09-10

**Authors:** Mei-Mei Zhao, Ting-Ting Ren, Jing-Kang Wang, Lu Yao, Ting-Ting Liu, Ji-Chao Zhang, Yang Liu, Lan Yuan, Dan Liu, Jiu-Hui Xu, Peng-Fei Tu, Xiao-Dong Tang, Ke-Wu Zeng

**Affiliations:** State Key Laboratory of Natural and Biomimetic Drugs, School of Pharmaceutical Sciences, Peking University, Beijing 100191, China; Beijing Key Laboratory of Musculoskeletal Tumor, Peking University People’s Hospital, Beijing 100044, China; State Key Laboratory of Natural and Biomimetic Drugs, School of Pharmaceutical Sciences, Peking University, Beijing 100191, China; State Key Laboratory of Natural and Biomimetic Drugs, School of Pharmaceutical Sciences, Peking University, Beijing 100191, China; State Key Laboratory of Natural and Biomimetic Drugs, School of Pharmaceutical Sciences, Peking University, Beijing 100191, China; State Key Laboratory of Natural and Biomimetic Drugs, School of Pharmaceutical Sciences, Peking University, Beijing 100191, China; Center of Basic Medical Research, Institute of Medical Innovation and Research, Peking University Third Hospital, Beijing 100191, China; Proteomics Laboratory, Medical and Healthy Analytical Center, Peking University Health Science Center, Beijing 100191, China; Proteomics Laboratory, Medical and Healthy Analytical Center, Peking University Health Science Center, Beijing 100191, China; Beijing Key Laboratory of Musculoskeletal Tumor, Peking University People’s Hospital, Beijing 100044, China; State Key Laboratory of Natural and Biomimetic Drugs, School of Pharmaceutical Sciences, Peking University, Beijing 100191, China; Beijing Key Laboratory of Musculoskeletal Tumor, Peking University People’s Hospital, Beijing 100044, China; State Key Laboratory of Natural and Biomimetic Drugs, School of Pharmaceutical Sciences, Peking University, Beijing 100191, China

**Keywords:** pyroptosis, ER membrane, antitumor immune, osteosarcoma, chemical degrader, reticulon-4 (RTN4)

## Abstract

Pyroptosis is an identified programmed cell death that has been highly linked to endoplasmic reticulum (ER) dynamics. However, the crucial proteins for modulating dynamic ER membrane curvature change that trigger pyroptosis are currently not well understood. In this study, a biotin-labeled chemical probe of potent pyroptosis inducer α-mangostin (α-MG) was synthesized. Through protein microarray analysis, reticulon-4 (RTN4/Nogo), a crucial regulator of ER membrane curvature, was identified as a target of α-MG. We observed that chemically induced proteasome degradation of RTN4 by α-MG through recruiting E3 ligase UBR5 significantly enhances the pyroptosis phenotype in cancer cells. Interestingly, the downregulation of RTN4 expression significantly facilitated a dynamic remodeling of ER membrane curvature through a transition from tubules to sheets, consequently leading to rapid fusion of the ER with the cell plasma membrane. In particular, the ER-to-plasma membrane fusion process is supported by the observed translocation of several crucial ER markers to the “bubble” structures of pyroptotic cells. Furthermore, α-MG-induced RTN4 knockdown leads to pyruvate kinase M2 (PKM2)-dependent conventional caspase-3/gasdermin E (GSDME) cleavages for pyroptosis progression. *In vivo*, we observed that chemical or genetic RTN4 knockdown significantly inhibited cancer cells growth, which further exhibited an antitumor immune response with anti-programmed death-1 (anti-PD-1). In translational research, RTN4 high expression was closely correlated with the tumor metastasis and death of patients. Taken together, RTN4 plays a fundamental role in inducing pyroptosis through the modulation of ER membrane curvature remodeling, thus representing a prospective druggable target for anticancer immunotherapy.

## Introduction

Endoplasmic reticulum (ER) is a crucial organelle that decides cell fates via the quality control of functional proteins (Hetz and Papa, 2018; Lin et al., 2007). A recent breakthrough in ER biology is the discovery of ER dysfunction as a critical molecular event in the occurrence of various programmed cell death including pyroptosis (Chen et al., 2019; Lebeaupin et al., 2015; Li et al., 2020; Zhang et al., 2021). Particularly, several studies have demonstrated a potential role of ER in triggering pyroptosis via NOD-like receptor protein 3 (NLRP3) inflammasome-mediated mechanism (Ke et al., 2020; Wang et al., 2020). Meanwhile, co-location of ER markers C/EBP homologous protein (CHOP) and immunoglobulin heavy chain binding protein (BIP) with pyroptosis markers has also been reported (Zeng et al., 2020). However, to date, the detailed biological mechanisms responsible for driving ER-associated pyroptosis have not been fully clarified (Kayagaki et al., 2021).

Numerous lines of evidence support the concept that pyroptotic cells form typical “bubble” structures by cell-surface remodeling (Yu et al., 2021). Since the maintenance of ER function necessitates an intact membrane system, the highly dynamic nature of the ER as an extensive membrane system suggests a fundamental potential in the aberrant cell membrane changes, such as the specific “bubble” membrane structures during pyroptosis. In particular, mechanistic studies have elucidated that ER membrane curvature intricately controls the transition of ER morphology from tubules to sheets, thereby facilitating ER stress response and the establishment of intracellular interactions with neighboring organelles, ultimately impacting cellular homeostasis (Puhka et al., 2012; Westrate et al., 2015). However, the precise molecular mechanism of ER membrane dynamics change during pyroptosis is currently not well understood. Meanwhile, the specific functional proteins that are engaged in remodeling ER membrane curvature to drive pyroptosis have yet to be fully characterized.

Reticulons (RTNs) are a specific class of crucial ER curvature stabilizing proteins encoded by four genes in mammals (RTN1-4) (Diekmann et al., 2005). RTNs mainly localize to ER tubules and the curved edges of ER sheets for cisternae structure stabilization (Breeze et al., 2016; Joshi et al., 2016; Shibata et al., 2010). Besides, RTNs have been reported to maintain ER network structure, protein secretion, and transporting ER constituents (Voeltz et al., 2006; Wu and Voeltz, 2021). Particularly, recent reports show that RTNs are widely involved in a range of cell fates determination such as apoptosis, autophagy, and inflammation response, hinting that RTNs may serve as a potential regulator in controlling crucial cellular physiological processes (Carter et al., 2022; Lorenzo et al., 2011; Xiao et al., 2022). Previous reports have shown that targeting RTN4 with chemical small molecules can impair ER and nuclear envelope morphology in cancer cells. However, it is not yet clear whether these compounds can induce cell pyroptosis by regulating ER membrane dynamics. Therefore, it is essential to conduct in-depth biological functional studies using specific small molecule probes targeting RTN4 to elucidate the precise role of RTN4 in the dynamics of the ER membrane changes during pyroptosis.

In this study, we first identified α-mangostin (α-MG) as a potent chemical inducer of cell pyroptosis. Then, we prepared the biotin-labeled probe for α-MG and revealed that ER-shaping protein RTN4 as a crucial cellular target in controlling pyroptosis progression via chemical genetics strategy. Unexpectedly, α-MG induces the degradation of RTN4 through a mechanism similar to molecular glue, recruiting the E3 ligase ubiquitin protein ligase E3 component N-recognin 5 (UBR5). Then, we found that chemical or genetic RTN4 knockdown significantly promoted dynamic ER membrane curvature remodeling via tubules-to-sheets transition. Of note, RTN4 deficiency significantly induced the ER-plasma membrane fusion to drive the typical “bubble” structures formation in pyroptotic cells. Furthermore, these observations were supported by the high expression of several ER markers in “bubble” structures. *In vivo*, RTN4 knockdown exerted a promising effect to activate antitumor immunity. Meanwhile, high RTN4 expression has been confirmed in individuals with osteosarcoma as potential therapeutic target or biomarker.

Collectively, our findings reveal that RTN4 contributes to progress of pyroptosis via morphological alterations of ER membrane curvature, which represents a fundamental target in drug development for anticancer therapeutics.

## Results

### Discovery of RTN4 as a functional protein in pyroptosis

**Figure 1.**
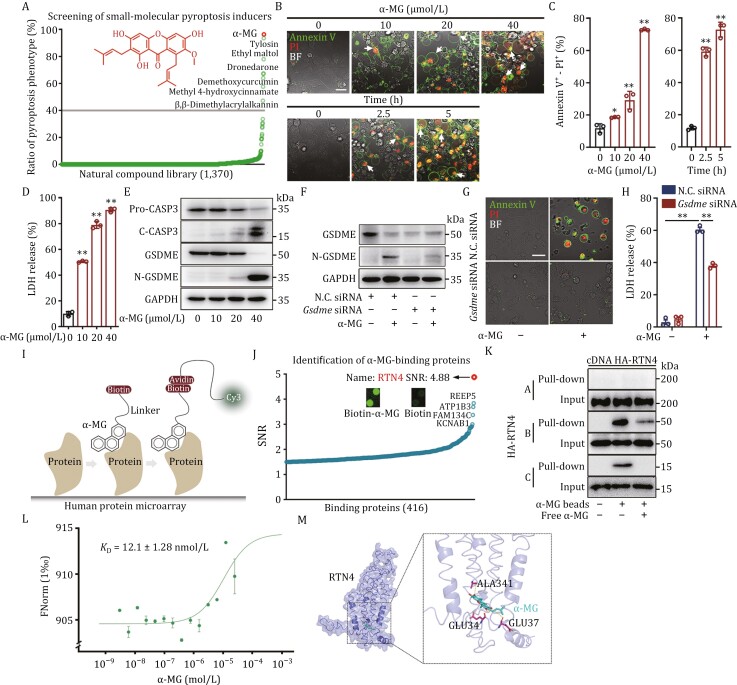
Discovery of RTN4 as a functional protein in pyroptosis. (A) High content screening of natural compound library containing 1,370 small molecules based on pyroptotic signatures in U2OS cells identified α-MG as an available chemical probe to induce cell pyroptosis. Ratio of pyroptosis phenotype (%) refers to the total number of cells with bubbles by the total number of cells. (B) α-MG-induced pyroptotic morphology as evidenced by Annexin V-PI double staining (scale bar: 25 μm). (C) α-MG increased the ratio of pyroptotic cells by Annexin V-PI flow analysis. (D) α-MG promoted the release of LDH. (E) α-MG-induced caspase-3 activation and GSDME cleavage by immunoblot assay. (F) GSDME expression was detected by immunoblot after GSDME knockdown. (G) GSDME knockdown inhibited α-MG-induced pyroptosis by Annexin V-PI double staining (scale bar: 25 μm). (H) LDH release assay confirmed that GSDME knockdown reversed α-MG-induced pyroptosis in U2OS cells. (I) Schematic diagram of α-MG-binding proteins identification with the human proteome microarray. (J) A total of 416 α-MG-binding proteins with SNR greater than 1.5 were identified. RTN4 had the highest SNR. (K) The interaction of α-MG with RTN4 isoforms was examined by competitive pull-down assay. (L) The binding of α-MG to RTN4 was determined by MST. (M) Molecular docking of α-MG towards RTN4. Data were presented as mean ± SD for three individual experiments. *P < 0.05, **P < 0.01 vs. control group.

Chemical genetics is a crucial strategy to discover functional proteins by using bioactive small molecules as chemical tools (Carlson and White, 2011; Gangadhar and Stockwell, 2007). Through pyroptosis phenotype-based high-content screening, we first identified α-mangostin (α-MG), a natural-derived xanthone derivative (Chen et al., 2018), as an available chemical probe to induce potent pyroptosis phenotype (Figs. 1A and S1A). Here, we selected osteosarcoma cells with the most prominent phenotype change for further study. We observed that α-MG concentration- and time-dependently increased the proportion of pyroptosis by Annexin V-PI double staining (Fig. 1B), flow quantitative analysis (Fig. 1C), and lactate dehydrogenase (LDH) release assay (Fig. 1D) in U2OS cells. Immunoblot analysis also confirmed that α-MG induced a typical cleavage of GSDME by caspase-3 (Fig. 1E), indicating an effective chemical tool for interrogating pyroptosis biology. Specifically, to investigate whether GSDME-mediated pyroptosis is the predominant mechanism of cell death induced by α-MG, we conducted knockdown experiments utilizing small interfering RNA (siRNA) to effectively suppress the expression of GSDME. As shown in Fig. 1F–H, silencing of GSDME resulted in a significant reversal of α-MG-induced pyroptotic bodies formation and LDH release, indicating a dependence on GSDME for pyroptosis.

Additionally, we detected the expression levels of GSDME in different osteosarcoma cells (U2OS, HOS, and 143B) and normal osteoblast cell line MC3T3, as well as clinical osteosarcoma samples. As shown in Fig. S1B, the expressions of GSDME in these osteosarcoma cells are significantly higher than the control MC3T3 cells, indicating that osteosarcoma potentially comprises a cellular subset demonstrating elevated GSDME expression levels. Meanwhile, the presence of GSDME levels was observed in clinical osteosarcoma samples, confirming the outcomes of cell culture investigations (Fig. S1B). Furthermore, we detected GSDME expression in paraffin-embedded specimens gathered from 102 patients with primary osteosarcoma by immunohistochemistry. The results revealed a high expression in osteosarcoma compared with para-tumor tissue. In particular, the high levels of GSDME in osteosarcoma tissues were positively associated with tumor metastasis and death of patients within 3 years (Fig. S1C–E). Meanwhile, the levels of caspase-3 in osteosarcoma tissues did not demonstrate significant differences in relation to tumor metastasis and patient mortality within a 3-year period (Fig. S1D and S1F). Given that caspase-3 and GSDME are two key factors involved in cell pyroptosis, we propose that caspase-3 may serve as a potential co-factor of GSDME in promoting pyroptosis in osteosarcoma cells. Therefore, GSDME can potentially serve as a risk assessment factor for investigating the onset of osteosarcoma, while caspase-3 can be utilized in conjunction for evaluation. Overall, these results indicate that α-MG tends to induce pyroptosis in osteosarcoma cells with high expression of GSDME.

Since α-MG has been previously known to inhibit IDH1-R132H (Kim et al., 2015), we established U2OS cells overexpressing IDH1-R132H and subsequently assessed the impact of α-MG on cell pyroptosis by Annexin V-PI double staining. We observed that overexpression of IDH1-R132H in U2OS cells did not result in any significant changes in the pyroptotic phenotype induced by α-MG, compared to wild-type control U2OS cells (Fig. S1G and S1H). In addition, we also employed the classic IDH1-R132H inhibitor AGI-5198 to treat U2OS cells in the presence of α-MG. Our result revealed that AGI-5198 did not significantly impact the pyroptotic phenotype induced by α-MG in U2OS cells, as assessed by Annexin V-PI double staining (Fig. S1I and S1J). Therefore, we propose that IDH1-R132H may not be the primary contributor to α-MG-induced cell pyroptosis in U2OS cells.

To explore the biological target of α-MG, we synthesized a biotin-labelled α-MG (biotin-α-MG, Fig. S2A–F) to discover potential binding proteins using HuProt proteome microarray, followed by incubation with Cy3-conjugated streptavidin (Fig. 1I). As a result, a protein with the highest signal to noise ratio (SNR) was identified as reticulon-4 (RTN4) (Fig. 1J). It is well known that RTN4 contains three major isoforms: RTN4A, RTN4B, and RTN4C, which are characterized by the critical regulator of ER curvature (Collins, 2006; Kiseleva et al., 2007; Kumar et al., 2021). Here, we found that RTN4B is the predominant isoform of RTN4 expressed in various tumors, especially in sarcoma by the cancer genome atlas (TCGA) database analysis (Fig. S1K). Next, we found that biotin-α-MG predominantly captured RTN4B and a small amount of RTN4C, using competitive pull-down analysis (Fig. 1K). Therefore, we focused on RTN4B for further investigation. The microscale thermophoresis (MST) assay demonstrated that both α-MG and biotin-α-MG directly bound to RTN4 (α-MG *K*_D_ = 12.1 ± 1.28 nmol/L, biotin-α-MG *K*_D_ = 39.0 ± 1.09 nmol/L) (Figs. 1L and S2G). Furthermore, molecular docking analysis revealed that α-MG exhibited spatial bonding around RTN4 through the formation of multiple hydrogen bonds with residues such as glutamate (GLU) 34, GLU 37, and alanine 341 (Fig. 1M). Similarly, biotin-α-MG is also bound to RTN4 through the establishment of several hydrogen bonds with GLU 34, GLU 37, and GLU 41 (Fig. S2H). Taken together, we identify RTN4 as a direct binding protein of α-MG that is highly associated with pyroptosis progression.

### RTN4 deficiency significantly promotes pyroptosis phenotype

**Figure 2.**
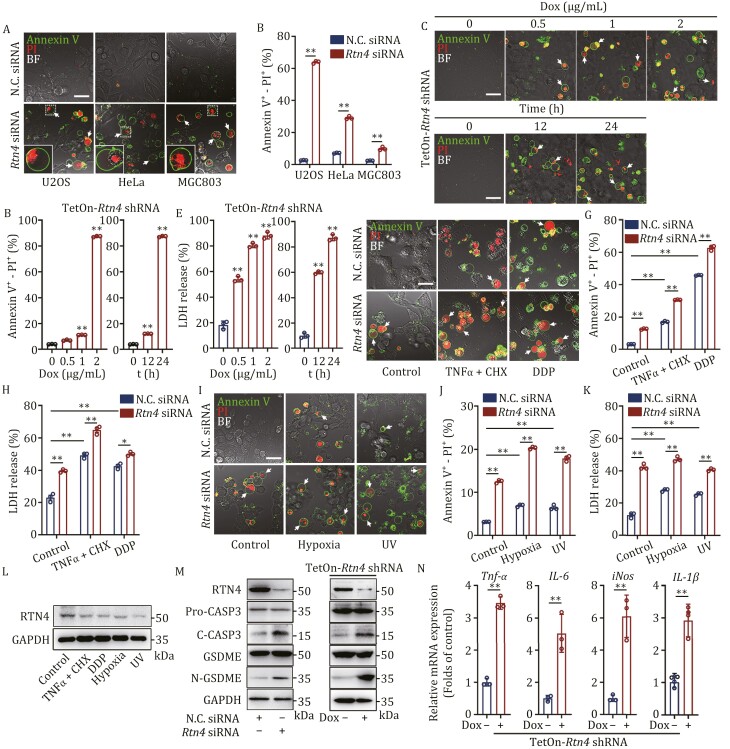
RTN4 deficiency significantly promotes pyroptosis phenotype. (A) RTN4 siRNA induced obvious pyroptosis morphology in U2OS, HeLa, and MGC803 by Annexin V-PI double staining (scale bar: 25 μm). (B) RTN4 siRNA increased the ratio of pyroptotic cells by Annexin V-PI flow analysis. (C) Doxycycline (dox)-mediated RTN4 downregulation induced pyroptosis morphology in U2OS cells by Annexin V-PI double staining (scale bar: 25 μm). (D) Dox-mediated RTN4 downregulation increased the ratio of pyroptotic cells by Annexin V-PI flow analysis. (E) LDH release was significantly elevated after dox treatment. (F–H) RTN4 siRNA treatment enhanced the pyroptosis phenotype in tumor necrosis factor-α (TNFα)/CHX or cisplatin (DDP)-treated U2OS cells by Annexin V-PI double staining (scale bar: 25 μm), quantitative flow and LDH release assay. (I, J, and K) Hypoxia and UV radiation showed synergetic effects with RTN4 siRNA on pyroptosis by Annexin V-PI double staining (scale bar: 25 μm), flow, and LDH release assay. (L) RTN4 expression was down-regulated in chemical or physical stress-treated U2OS cells. (M) RTN4 knockdown activated caspase-3 to cleave GSDME. (N) RAW264.7 cells exhibited a significant activation by the supernatants from U2OS cells with RTN4 knockdown. The expressions of TNF-α, interleukin-6 (IL-6), inducible nitric oxide synthase (iNOS), and IL-1β were determined by qPCR assay. Data were presented as mean ± SD for three individual experiments. *P < 0.05, **P < 0.01 vs. control group.

To determine whether RTN4 plays a role in pyroptosis progression, we performed RTN4 silencing in osteosarcoma cell lines (U2OS, HOS, and 143B) and observed significant pyroptosis-associated “bubble” morphology by Annexin V-PI double staining (Figs. 2A and S3A). Similar phenotypes were also observed in other cell lines such as HeLa and MGC803 (Fig. 2A). Moreover, flow-cytometry analyses showed that RTN4 siRNA significantly elevated the pyroptosis percentage of Annexin V-PI positive cells (Figs. 2B and S3B). We next constructed a lentiviral miR30-based Tet-inducible (pLKO-Tet-On) short hairpin RNA (shRNA) system to specially knock down RTN4 in U2OS cells (Fig. S3C and S3D). As shown in Fig. 2C, doxycycline (dox) significantly induced typical pyroptotic “bubble” morphology in U2OS cells by Annexin V-PI double staining (Fig. 2D). Also, dox treatment increased LDH release in U2OS cells (Fig. 2E), suggesting that RTN4 deficiency highly contributes to pyroptosis progression.

Next, we explored the synergic effect of RTN4 knockdown with various cell stress stimulations. We observed that RTN4 knockdown sensitized the pyroptosis phenotype in TNF-α/cycloheximide (CHX) (Hu et al., 2020) or cis-platinum (DDP) (Wang et al., 2017) -treated U2OS cells (Fig. 2F). Meanwhile, HeLa and MGC803 cells exhibited heightened susceptibility to pyroptosis following RTN4 knockdown (Fig. S3E). These observations were further confirmed by quantitative flow-cytometry measurement (Fig. 2G) and LDH release assay (Fig. 2H). Subsequently, physical stimulations such as hypoxia (Yu et al., 2019) and UV radiation (Liu et al., 2021) also showed synergetic effects on pyroptosis induction in the presence of RTN4 knockdown (Figs. 2I–K and S3F). Interestingly, both chemical and physical stress downregulated the expression of RTN4, which represented a universal biological response during pyroptosis (Fig. 2L). Furthermore, immunoblot assay revealed that RTN4 deficiency dramatically activated caspase-3 to cleave GSDME (Figs. 2M and S3G), and subsequently prompted a strong immune response in RAW264.7 macrophages by enhancing inflammatory gene expressions of TNF-α, IL-6, iNOS, and IL-1β (Figs. 2N and S3H). Finally, we performed a rescue experiment in TetOn-RTN4 shRNA U2OS stable cell lines to elucidate the isoform of RTN4 involved in mediating pyroptosis. Remarkably, our results presented in Fig. S3I revealed that the overexpression of RTN4B effectively restored caspase-3 and GSDME cleavage, whereas the transfection of RTN4A or RTN4C plasmids did not induce a comparable effect. Collectively, these findings strongly suggest that RTN4B plays a pivotal role in the regulation of pyroptosis.

### α-MG serves as a RTN4 degrader by recruiting E3 ligase UBR5 in ubiquitin-proteasome system

**Figure 3.**
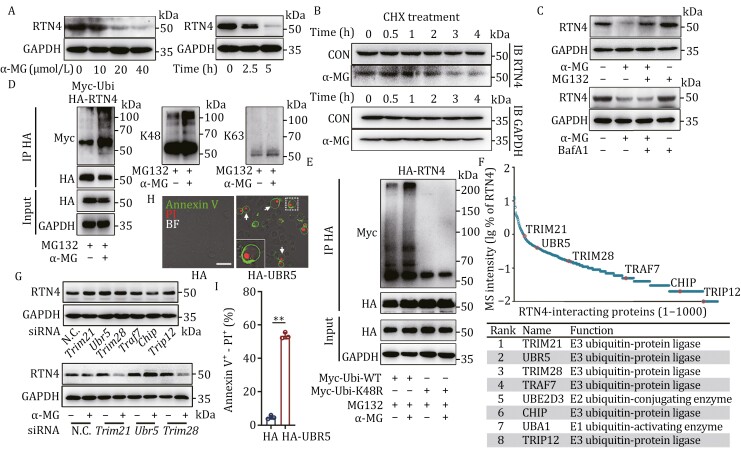
**α**-MG serves as a RTN4 degrader by recruiting E3 ligase UBR5 in ubiquitin-proteasome system. (A) α-MG downregulated RTN4 expression in a concentration- and time-dependent manner by immunoblot assay. (B) α-MG downregulated RTN4 expression in the presence of CHX at different time points. (C) Proteasome inhibitor MG132 but not lysosome inhibitor Bafilomycin A1 reversed α-MG-induced RTN4 degradation by western blot. (D) α-MG promoted the K48-linked but not the K63-linked ubiquitination of RTN4. (E) Transfection of K48-resistant ubiquitin (Lys48 to Arg48 mutant, K48R) reversed the ubiquitination of RTN4. (F) Co-IP coupled with MS identified RTN4-interacting protein components. The table showed representative ubiquitin-protein ligases identified by MS. (G) The impact of E3 ligase knockdown on RTN4 degradation in response to α-MG treatment. (H and I) The influence of UBR5 on pyroptosis in U2OS cells was characterized by Annexin V-PI double staining (scale bar: 25 μm). **P < 0.01 vs. control group.

Next, we examined the effect of α-MG on the expression of RTN4. Interestingly, we observed that α-MG significantly reduced RTN4 protein expression in a concentration- and time-dependent manner (Figs. 3A and S4A), without affecting mRNA levels (Fig. S4B). Then, we used protein synthesis inhibitor cycloheximide (CHX) to block RTN4 protein synthesis and found that RTN4 expression was also downregulated upon α-MG treatment, suggesting that α-MG functions as a RTN4 chemical degrader (Fig. 3B). Subsequent treatment with MG132 and Bafilomycin A1 (BafA1) indicated that α-MG predominantly degraded RTN4 through the proteasome system rather than the lysosomal pathway (Fig. 3C).

Given that the degradation of RTN4 primarily relies on the proteasomal pathway, we next investigated whether RTN4 undergoes ubiquitination modification. Western blot analysis revealed that α-MG significantly promoted the ubiquitination modification of RTN4 (Fig. 3D). In particular, this ubiquitination modification was mainly mediated by K48-dependent ubiquitin chain formation, with little impact on K63 ubiquitin chain formation (Fig. 3E). We then hypothesized that α-MG facilitated K48-dependent ubiquitination modification of RTN4 by recruiting specific E3 ligases. Therefore, we immunoprecipitated HA-tagged RTN4 after MG132 treatment and identified the interacting proteins using mass spectrometry (MS) (Fig. 3F). Then, we identified a total of 1,290 intracellular proteins that interact with RTN4. Upon refining the key ubiquitin ligases of RTN4, we finally identified six E3 ligases, including TRIM21, UBR5, TRIM28, TRAF7, CHIP, and TRIP12 (Fig. 3F). Meanwhile, we also identified the E1 ubiquitin-activating enzyme UBA1 and the E2 ubiquitin enzyme UBE2D3 (Fig. 3F). To further determine which E3 ubiquitin ligase is involved in the ubiquitination of RTN4, we knocked down the expression of these E3 ubiquitin ligases in cells using siRNA. Then, we treated the cells with α-MG and found that the degradation of RTN4 induced by α-MG was only reversed upon UBR5 knockdown (Fig. 3G). Moreover, UBR5 knockdown effectively hindered α-MG-induced pyroptotic bodies formation (Fig. S4C). Additionally, we observed that overexpression of UBR5 induced a “bubble” morphology in U2OS cells, as assessed by Annexin V-PI double staining and quantified via flow-cytometry analysis (Fig. 3H and 3I). Thus, we concluded that α-MG likely promoted the molecular glue-like interaction between UBR5 and RTN4, subsequently enhancing the downstream proteasomal degradation of RTN4 via the K48-dependent pathway.

### RTN4 knockdown remodels ER membrane curvature via tubule-to-sheet change

**Figure 4.**
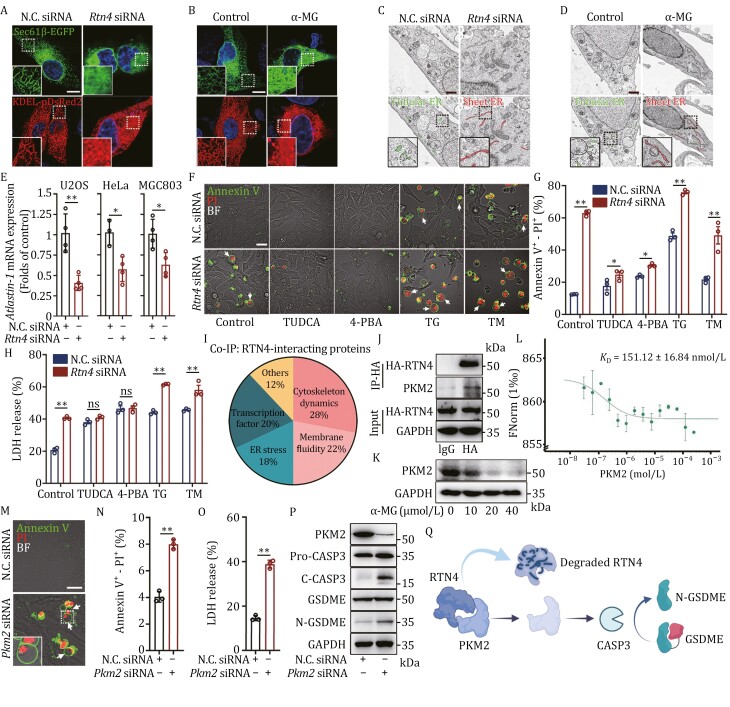
RTN4 knockdown remodels ER membrane curvature via tubule-to-sheet change. (A and B) RTN4 knockdown or α-MG-induced obvious ER tubules-to-sheets morphology in U2OS cells expressing Sec61β-EGFP and KDEL-pDsRed2 by Airyscan super-resolution microscopy (scale bar: 10 μm). (C and D) TEM imaging of tubular ER (less 10 μm in length) and sheet ER (over 10 μm in length) in RTN4 siRNA or α-MG-treated U2OS cells (scale bar: 10 μm). (E) Atlastin-1 mRNA expression was downregulated in RTN4-silenced U2OS, HeLa, and MGC803 cells by qPCR analysis. (F) ER stress inhibitors TUDCA or 4-PBA reversed RTN4 knockdown-induced pyroptosis morphology; meanwhile, ER stress inducers TG or TM enhanced the “bubble” morphology in U2OS cells by Annexin V-PI double staining (scale bar: 25 μm). (G) Quantitative analysis of Annexin V-PI double positive pyroptotic cells by flow cytometry. (H) LDH release analysis of ER stress inhibitors or inducers-treated U2OS cells upon RTN4 knockdown. (I) RTN4-interacting proteins were enriched via Co-IP assay in U2OS cells and classified into 5 function-based groups. (J) RTN4 interacted with PKM2 by co-IP assay from the lysates of HA-RTN4 overexpressed-U2OS cells. (K) α-MG exhibited a concentration-dependent decrease in PKM2 protein expression. (L) The dissociation constant (*K*_D_) between RTN4 with PKM2 was measured as 151.12 ± 16.84 nmol/L by MST. (M, N, and O) Annexin V-PI double staining (scale bar: 25 μm), quantitative flow and LDH release assay confirmed that PKM2 knockdown triggered pyroptosis phenotype in U2OS cells. (P) PKM2 knockdown triggered caspase-3/GSDME pathway. (Q) Schematic of the RTN4-mediated pyroptosis mechanism. RTN4 degradation results in the release of its interacting protein, PKM2, which triggers the activation of the PKM2-mediated caspase-3/GSDME signaling pathway. Data were presented as mean ± standard deviation (SD) for three individual experiments. *P < 0.05, **P < 0.01 vs. control group.

Since RTN4 functions as a pivotal protein in regulating ER membrane curvature, thus RTN4 deficiency is likely to impact the morphological characteristics of ER (Kumar et al., 2021). Here, confocal fluorescence analysis revealed that RTN4 knockdown by siRNA induced ER morphology alteration from grid-like tubules to flattened sheet-like structures in U2OS cells, which were transfected with ER markers Sec61β-EGFP and Lys-Asp-Glu-Leu (KDEL)-pDsRed2 (Fig. 4A). Moreover, we observed that chemical-induced RTN4 degradation with α-MG also markedly triggered ER tubules-to-sheets transition (Fig. 4B). Furthermore, we performed transmission electron microscope (TEM) analysis to confirm the marked increases in the proportion of sheet ER over 10 μm in length both in RTN4 siRNA and α-MG-treated U2OS cells (Fig. 4C and 4D). Since atlastin-1 serves as a critical mediator of tubular ER formation, we also performed quantitative real-time PCR (qPCR) analysis and found that the mRNA expression of atlastin-1 was significantly downregulated in RTN4-silenced U2OS cells, as well as HeLa and MGC803 (Fig. 4E), indicating that ER tubules-to-sheets transition was highly associated with the intracellular expression of RTN4.

Recent evidence indicates that RTN4 morphological imbalance potentially induces ER stress (Kuang et al., 2006; Oertle et al., 2003). We then performed Annexin V-PI double staining and observed that ER stress inhibitors tauroursodeoxycholic acid (TUDCA) or 4-phenylbutiric acid (4-PBA) reversed RTN4 knockdown-induced “bubble”-like pyroptosis phenotype (Fig. 4F). Meanwhile, ER stress inducers thapsigargin (TG) or tunicamycin (TM) significantly enhanced the “bubble” morphology in U2OS cells (Fig. 4F). These findings were also confirmed by flow cytometry (Fig. 4G) and LDH release assay (Fig. 4H), indicating a critical bridging function of ER stress in RTN4 deficiency-induced pyroptosis. Next, to gain a detailed insight into the relationship between RTN4 and ER morphology transition, we performed co-immunoprecipitation (co-IP) experiment to capture the RTN4-binding proteins in cells. Then, 96 proteins were significantly enriched, and classified into five classes, including cytoskeleton dynamics, membrane fluidity, transcription factor, ER stress, and others (Fig. 4I). Among these RTN4-binding proteins, the pyruvate kinase M2 (PKM2) especially attracted our interest. Since PKM2 has previously been reported to be highly associated with pyroptosis progress via regulating ER stress (Li et al., 2021a, 2021b), and thus considered as a crucial candidate protein for further investigation. To test the interaction of RTN4 with PKM2, we performed co-IP assay and found that RTN4 is directly bound to PKM2 (Fig. 4J). In parallel, the treatment of α-MG led to a downregulation of PKM2 expression, ultimately resulting in the cleavage of GSDME (Fig. 4K). Furthermore, MST analysis to quantificationally characterize the affinity of RTN4 with PKM2 revealed the dissociation constant (*K*_D_) as 151.12 ± 16.84 nmol/L (Fig. 4L), demonstrating a potent interaction between RTN4 and PKM2. Furthermore, PKM2 knockdown prompted “bubble” morphology in U2OS cells that was characterized by Annexin V-PI double staining (Fig. 4M and 4N). Meanwhile, PKM2 knockdown facilitated LDH release, eventually triggering pyroptosis by inducing caspase-3/GSDME activation (Fig. 4O–Q). Next, to investigate the influence of PKM2 on ER membrane curvature, we conducted confocal fluorescence analysis in U2OS cells expressing the ER marker Sec61β-EGFP. PKM2 knockdown failed to induce ER tubules-to-sheets transition (Fig. S5A). Furthermore, we observed UBR5 overexpression-induced ER morphology alteration from grid-like tubules to flattened sheet-like structures (Fig. S5B). Meanwhile, knockdown of UBR5 by siRNA had no obvious effect on ER membrane curvature alteration (Fig. S5C). Thus, these findings suggest that UBR5 but not PKM2 is an upstream signaling pathway regulating ER membrane curvature remodeling.

Finally, we conducted experiments to delve deeper into the correlation between ER membrane curvature remodeling and caspase-3/GSDME signaling. Our results revealed that the inhibition of caspase-3 using Z-DEVD-FMK or the depletion of GSDME through siRNA did not produce any noticeable impacts on the curvature of ER membrane such as the “tubule-to-sheet” transition (Fig. S5D and S5E). These findings suggest that caspase-3/GSDME signaling may potentially be situated downstream of ER membrane curvature remodeling.

In summary, our findings suggest that RTN4 deficiency-induced ER membrane curvature change results in pyroptosis via ER stress signaling pathway.

### Membrane curvature change drives ER fusion to the “bubble” structures during pyroptosis

**Figure 5.**
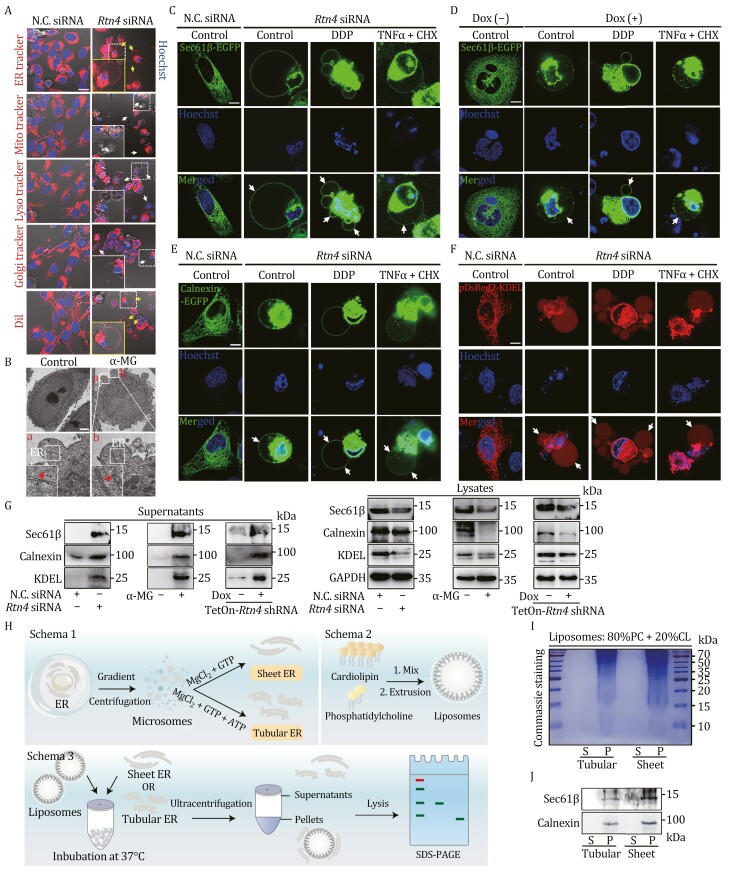
RTN4 knockdown promotes ER-membrane fusion to the “bubble” structures. (A) Fluorescence probes (ER tracker, Mito tracker, Lyso tracker, Golgi tracker, and Cell membrane tracker DiI) tracking of the “bubble” structures (scale bar: 25 μm). (B) α-MG-induced ER translocation onto the “bubble” structures as shown by TEM imaging (scale bar: 10 μm). (C and D) Sec61β-EGFP-labelled ER membrane translocated onto the “bubble” structures in RTN4 siRNA-treated U2OS cells or dox-inducible RTN4 knockdown model, which was enhanced by TNFα/CHX or DDP. (E and F) Calnexin-EGFP-labelled ER membrane or pDsRed2-KDEL-labelled ER lumen translocated to the “bubble” structures in RTN4 siRNA-treated U2OS cells, which was enhanced by TNFα/CHX or DDP. (G) ER marker proteins like Sec61β, Calnexin, and KEDL were secreted to supernatants upon RTN4 downregulation by immunoblot assay. (H) Schematic representation of the liposome-binding assay. Purified ER proteins were incubated with liposomes with 80% phosphatidylcholine and 20% cardiolipin. After ultracentrifugation, the liposome-free supernatants and the liposome pellets were analyzed by SDS-PAGE and Coomassie staining. (I) Liposomes preferred to bind with sheet ER rather than tubular ER. S: supernatants; P: pellets. (J) ER membrane proteins (Sec61β and Calnexin) were highly expressed at liposome-binding sheet ER constituents by immunoblot assay.

The “bubble” structure formation is a crucial biological event in pyroptosis (Chen et al., 2016). To explore the role of ER in RTN4 deficiency-induced “bubble”-like phenotype, we applied a series of fluorescent probes for cell organelles staining (ER tracker, Mito tracker, Lyso tracker, Golgi tracker, and Cell membrane tracker DiI). Interestingly, we found that RTN4-knockdown-induced “bubble” structures were obviously labelled by ER tracker, but not Mito tracker or Lyso tracker; while the bubbles were mildly labelled by Golgi tracker and DiI (Fig. 5A). Accordingly, we also confirmed this finding by RTN4 degrader α-MG (Fig. S6A). α-MG treatment resulted in the presence of ER in the “bubble” structures by TEM imaging (Fig. 5B). Next, to confirm the direct connection between ER and the “bubble” structures, we overexpressed ER markers (Sec61β, Calnexin, KDEL) in U2OS cells. Living cell imaging analysis showed that upon RTN4 knockdown with siRNA, the green fluorescence from Sec61β-EGFP appeared in the “bubble” structures (Fig. 5C). Similar results were also confirmed in dox-inducible RTN4 knockdown model (Fig. 5D). Moreover, Calnexin-EGFP-labelled ER membrane and pDsRed2-KDEL-labelled ER lumen were also highly expressed in the “bubble” structures of RTN4 knockdown cells (Fig. 5E and 5F). RTN4 degrader α-MG phenocopied the genetic RTN4 knockdown in the translocation of these ER markers to the “bubble” structures by fluorescence confocal (Fig. S6B and S6C). Furthermore, RTN4 silencing markedly prompted Sec61β-EGFP-labelled ER membrane co-location with GSDME-N pores around the “bubble” structures (Fig. S6D). Meanwhile, RTN4 degrader α-MG exhibited a similar effect to induce Sec61β-labelled ER membrane translocation (Fig. S6E).

In addition, immunoblot assay showed that ER proteins (Sec61β, Calnexin, and KDEL) were released from cytosol to culture supernatants in response to RTN4 knockdown (Fig. 5G), further supporting our assumption that ER may translocate to the “bubble” structures during pyroptosis. Of note, liposome-binding assay demonstrated that the proteins from purified sheet ER rather than tubular ER showed a binding preference for liposomes that resembled intracellular membranes (Figs. 5H, 5I and S6F). Moreover, ER membrane proteins including Sec61β and Calnexin were mostly found in pellets composed of sheet ER constituents binding with liposomes (Fig. 5J). Taken together, these data demonstrate that ER is highly involved in pyroptosis progression by promoting its membrane fusion to the “bubble” structures during pyroptosis.

### Plasma membrane disturbance synergistically promotes RTN4 deficiency-induced pyroptosis

**Figure 6.**
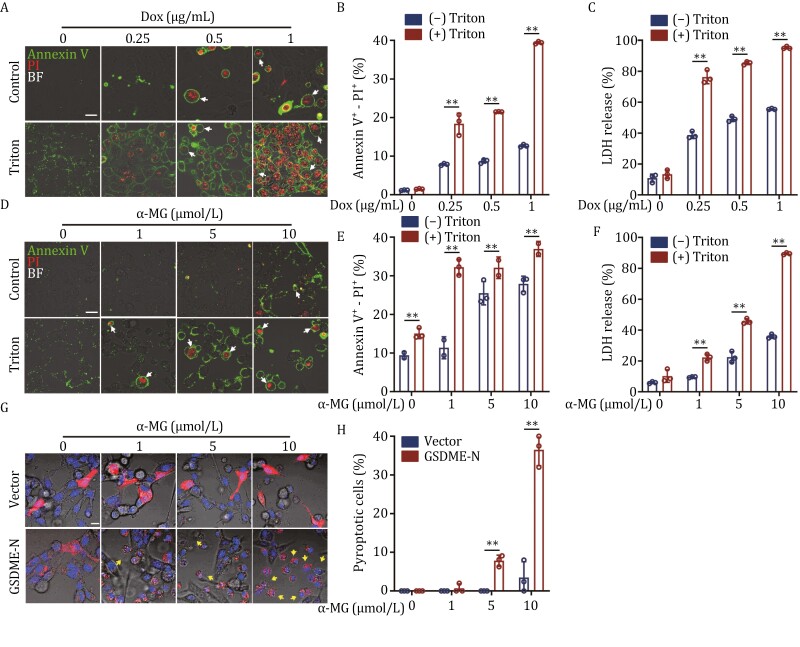
Plasma membrane disturbance synergistically promotes RTN4 deficiency-induced pyroptosis. (A and B) Triton X-100 (0.01%) upregulated the proportion of dox-induced “bubble” structures by Annexin V-PI double staining (scale bar: 25 μm) and flow analysis. (C) Triton X-100 significantly promoted the LDH release in dox-induced U2OS cells. (D) Triton X-100 synergistically promoted the “bubble” structures formation in α-MG-induced U2OS cells by Annexin V-PI double staining (scale bar: 25 μm). (E and F) Triton X-100 synergistically promoted pyroptosis in U2OS cells by flow analysis and LDH release assay. (G) GSDME-N overexpression with a mCherry tag enhanced α-MG-induced “bubble” structures formation (scale bar: 25 μm). (H) GSDME-N overexpression upregulated α-MG-induced proportion of pyroptotic cells. Data were presented as mean ± SD for three individual experiments. **P < 0.01 vs. control group.

Since ER membrane may contribute to constructing the “bubble” structures by ER-plasma membrane fusion, we then speculated that plasma membrane disturbance may affect pyroptosis progress. To this end, we used amphiphilic surfactant Triton X-100 to mildly treat the cells for partly removing Triton X-100-soluble plasma membrane constituents (Adachi et al., 2007). As shown in Fig. 6A, Triton X-100 alone did not trigger obvious pyroptosis in U2OS cells; however, Triton X-100 greatly upregulated the proportion of dox-induced “bubble” structures by Annexin V-PI double staining and flow analysis (Fig. 6B). Moreover, Triton X-100 also significantly promoted the LDH release in dox-induced U2OS cells (Fig. 6C). Similarly, Triton X-100 accelerated the “bubble” structures formation in RTN4 degrader α-MG-induced U2OS cells (Fig. 6D–F). Furthermore, overexpression of GSDME-N enhanced α-MG-induced “bubble” structures formation in pyroptosis (Fig. 6G and 6H). Collectively, these results indicate that plasma membrane homeostasis plays an essential role in promoting the “bubble” structures formation during RTN4 knockdown-induced pyroptosis.

### Translational study of RTN4 degrader for anticancer therapy

**Figure 7.**
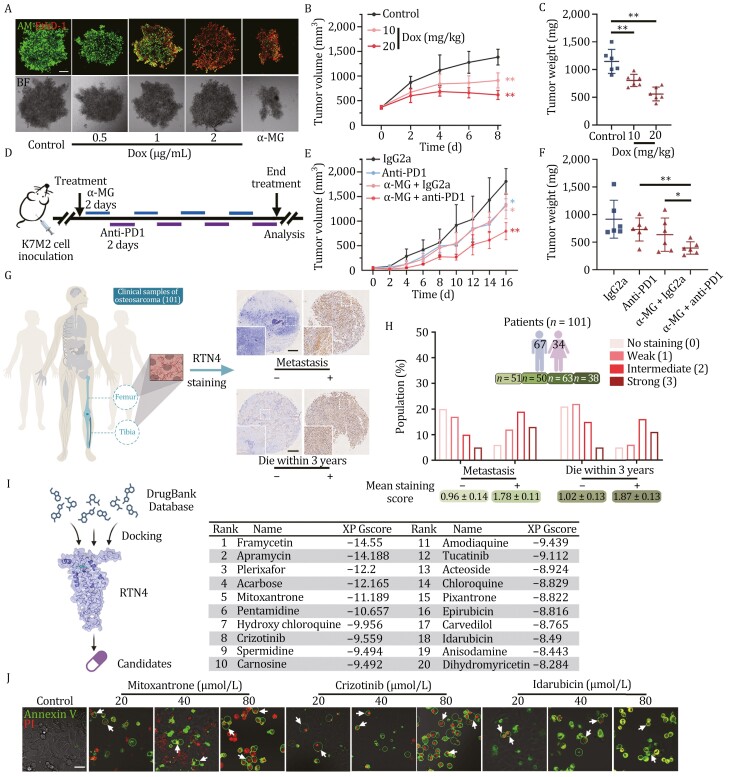
Translational study of RTN4 degrader for anticancer therapy. (A) 3D U2OS tumor spheroids were stained by Calcein-AM and EthD-1 at different concentrations of Dox (0.5, 1, and 2 μg/mL) for 24 h. RTN4 degrader α-MG (40 μmol/L) was also added for 3 h to induce tumor spheroid disintegration. Scale bar: 100 μm. (B and C) Intratumor injection of dox into the K7M2 osteosarcoma xenograft model containing a dox-inducible RTN4 shRNA lentivirus vector retarded tumor growth and downregulated the tumor weights. (D) Procedure of a combination scheme with α-MG and anti-PD-1 antibody. (E and F) α-MG administration coupled with anti-PD1 treatment displayed a marked synergistic action in reducing tumor growth and weights. (G) Detection of RTN4 expression on osteosarcoma chip containing 101 clinical samples. Scale bar: 200 μm. (H) Immunohistochemical staining scoring of RTN4 expression based on whether the tumors metastasized and the patients died within 3 years. Data were presented as mean ± SD (*n* = 6). *P < 0.05, **P < 0.01 vs. control group. (I) Virtual screening of RTN4-targeting small molecules. (J) Mitoxantrone, crizotinib, and idarubicin concentration-dependently increased the proportion of pyroptosis by Annexin V-PI double staining (scale bar: 25 μm).

To evaluate the antitumor potential of RTN4-targeting strategy, we established a 3D tumor spheroids model stained by Calcein-AM and EthD-1 (Kamoshima et al., 2011). As shown in Fig. 7A, dox exhibited a concentration-dependent antitumor activity in inhibiting the growth of 3D tumor models. RTN4 degrader α-MG also showed a similar result (Fig. 7A). Next, we established an osteosarcoma xenograft mouse model using the K7M2 cells expressing RTN4 shRNA activated by dox. We found that intratumor injection of dox efficiently impaired tumorigenesis via downregulation of RTN4 expression (Figs. 7B, 7C, S7A and S7B). Next, to explore the clinical transformation feasibility of RTN4 degradation for anticancer therapy, we tested the therapeutic effect of the oral administration of RTN4 degrader α-MG on K7M2-bearing mice. We observed that α-MG (50 or 100 mg/kg) significantly decreased the sizes (Fig. S7C and S7D) and weights (Fig. S7E) of K7M2 tumors, without body weight reduction (Fig. S7F). Moreover, immunohistochemistry (IHC) analysis showed that α-MG strongly upregulated T cell marker CD8, NK cell marker CD56, as well as macrophages marker F4/80 levels in tumor tissues (Fig. S7G), indicating an obvious effect of activating antitumor immunity. Meanwhile, we found that programmed cell death 1 ligand 1 (PD-L1) expression was significantly upregulated upon α-MG treatment (Fig. S7G), suggesting a synergistic possibility of α-MG with immune checkpoint therapy. We then designed a combination scheme with α-MG and anti-PD-1 antibody (Fig. 7D). Our result showed that α-MG administration coupled with anti-PD-1 treatment displayed a marked synergistic action in reducing tumor volumes and weights (Figs. 7E, 7F and S7H), without obvious adverse effects on body weights, organ weights, and serum biochemical indicators (Fig. S7I–L).

Furthermore, we detected RTN4 expression in paraffin-embedded specimens gathered from 101 patients with primary osteosarcoma by immunohistochemistry. The results revealed that high levels of RTN4 in osteosarcoma tissues as characterized by strong staining were positively associated with tumor metastasis and death of patients within 3 years via the manual scoring method (Figs. 7G, 7H and S8). In addition, we evaluated the immunohistochemical features of RTN4 in osteosarcoma tissues and normal bone tissues, which indicated a relatively high expression of RTN4 present in osteosarcoma tissues (Fig. S7M). Therefore, these results indicate that RTN4 may represent an available biomarker or therapeutic target for anticancer in translational medicine research.

Given the functional significance of RTN4 in pyroptosis as well as osteosarcoma progression, the inhibition of RTN4 represents a potential strategy for tumor therapy. To this end, we conducted a high-throughput virtual screening using the DrugBank Database, which comprises 10,845 compounds. Each compound was docked into the predicted binding pocket of the RTN4 structure generated by I-TASSER. Subsequently, Glide XP GScore values (kcal/mol), representing the binding affinities of these compounds to RTN4, were calculated. From this screening, the top 20 compounds (Fig. 7I) were selected for further experimental validation. Using the MST assay, we confirmed that a majority of these compounds exhibited strong binding affinity to RTN4 (Fig. S7N). Additionally, the characteristic analysis of pyroptosis in U2OS cells under bright-field microscopy as well as Annexin V-PI double staining revealed that Mitoxantrone, Crizotinib, and Idarubicin markedly induced pyroptosis (Figs. S7O, S7P and 7J). Taken together, we have demonstrated the potential of targeting RTN4 to induce pyroptosis, thereby providing lead compounds for the development of RTN4-targeted therapeutic agents.

## Discussion

Accumulating studies have shown that pyroptosis, a newly discovered programmed cell death, undergoes a unique biological membrane reconstruction process to produce “bubble” structures (Rühl et al., 2018; Wang et al., 2021). During this process, ER has been reported to function as a potential driving force to promote pyroptosis progression (Tall and Westerterp, 2019), however, the detailed biological mechanisms are still missing. In this study, we discovered that ER-shaping protein RTN4 exerted a fundamental function in promoting the “bubble” structures formation during pyroptosis. Moreover, chemical or genetic knockdown of RTN4 significantly induced ER tubules-to-sheets change to accelerate ER stress and subsequent GSDME cleavage. More importantly, RTN4 knockdown regulated ER membrane curvature to promote contact and fusion with the plasma membrane, thereby potentially providing basic materials for building the “bubble” structures during pyroptosis.

As an important organelle in eukaryotic cells, ER is a continuous membrane system that forms the interconnected network of tubules and sheets (Shibata et al., 2006). Here, our results provide an assumption that ER tubules-to-sheets change facilitates ER movement along microtubule cytoskeleton to GSDME membrane pores, hinting an interesting clue that ER provides raw biological materials for the “bubble” structures formation during pyroptosis. Given the pyroptotic cells generally produce the “bubble” structures within a short time (e.g., within 2 h), a feasible strategy for driving this progress is the efficient utilization of existing resources of ER membrane system, which serves as the largest continuous membrane-bound compartment in eukaryotic cells (Rühl et al., 2018). Thus, we propose that cells may have evolved a potential ability to restructure biological membrane structure via reutilizing ER membrane materials (biology membrane economy). Although more measurements are needed to promote the confirmation of this hypothesis, our current findings may provide initial evidence to broaden our understanding of the function complexity and fast-response mechanism in pyroptosis progress.

As for the molecular mechanism how ER membrane dynamics mediates the “bubble” structures formation, one possible explanation for the initial stage may be that ER membranes fuse into plasma membrane, and are extruded out of gasdermin pores under osmotic pressure to form bubble-like structures (pre-pyroptotic bodies) (Mamun et al., 2024; Zheng et al., 2024). In fact, our observations support this speculation that several ER membrane proteins were identified on the pyroptotic bubbles including Sec61β, Calnexin, and KDEL. Moreover, we found that a small amount of Golgi markers was also expressed on the bubbles, suggesting that Golgi apparatus may be partly involved in the progress of plasm membrane blebbing via ER-Golgi trafficking. Moreover, another explanation may underline the importance of RTN4/PKM2 signaling in activating the cleavage of caspase 3 and GSDME, thereby contributing to a driving force in triggering pyroptosis punching.

Pyroptosis plays a pivotal role in reshaping the tumor immune microenvironment, thereby triggering robust antitumor immune responses. The specific benefit of RTN4-mediated pyroptosis in enhancing the efficacy of antitumor immune therapy lies in its capacity to increase the expression of PD-L1 in tumor cells and subsequently induce immune-mediated tumor cell death. The dual mechanism of attracting PD-L1-expressing tumor cells followed by selective elimination significantly enhances the therapeutic outcomes in tumor therapy. Currently, a major challenge for anticancer therapy via targeting pyroptosis suffers from a serious lack of available small molecules (Loveless et al., 2021; Wang et al., 2017). With α-MG as a specific RTN4 chemical degrader, we found that α-MG obviously decreased RTN4 expression for promoting ER tubules-to-sheets transition, subsequently causing pyroptosis in cancer cells. This observation further supported that RTN4 may function as a valuable drug target for anticancer by controlling ER dynamics in pyroptosis, indicating potential benefits in medical translation. Meanwhile, to the best of our knowledge, α-MG is the first small molecule targeting RTN4 to enhance pyroptosis-based antitumor immune responses. Specifically, α-MG may serve as a molecular glue to induce the RTN4 degradation by recruiting E3 ligase UBR5. Therefore, α-MG may provide a novel molecular templet for drug candidate development for anticancer via activating the function of ubiquitin-proteasome system.

In summary, we show that RTN4 is highly involved in driving pyroptosis via dynamic remodeling ER membrane curvature. These findings may provide an attractive direction for our understanding of the fundamental ER biology during pyroptosis, and underline the importance of RTN4 as a valuable anticancer target.

## Supplementary data

Supplementary data is available at *Protein & Cell* online at https://doi.org/10.1093/procel/pwae049.

pwae049_suppl_Supplementary_Figures_S1-S8

## Data Availability

The main data supporting the results in the study are available within the paper and its Supplementary Information. The raw and processed datasets generated during the current study are available for research purposes from the corresponding authors upon reasonable request.

## Abbreviations

4-PBA: 4-phenylbutiric acid
α-MG: α-mangostin
ALA: alanine
BafA1: Bafilomycin A1
co-IP: co-immunoprecipitation
dox: doxycycline
CHX: cycloheximide
ER: endoplasmic reticulum
GLU: glutamate
IHC: immunohistochemistry
LDH: lactate dehydrogenase
Lyso: lysosome
Mito: mitochondria
MST: microscale thermophoresis;
PKM2: pyruvate kinase M2
qPCR: quantitative polymerase chain reaction;
RTN4: reticulon-4
SARC: sarcoma
SD: standard deviation
shRNA: short hairpin RNA
siRNA: small interfering RNA
SNR: signal to noise ratio
TG: thapsigargin
TM: tunicamycin
TNF-α: tumor necrosis factor-α
TUDCA: tauroursodeoxycholic acid
