# Supplementary material for: Endoplasmic reticulum membrane remodeling by targeting reticulon-4 induces pyroptosis to facilitate antitumor immune
Source: Protein Cell. 2024 Sep 10;16(2):121–35. doi: 10.1093/procel/pwae049 (PMC11786723; doi:10.1093/procel/pwae049)
Supplement: pwae049_suppl_Supplementary_Figures_S1-S8 [file pwae049_suppl_supplementary_figures_s1-s8.pdf]

Supplemental information for

ER membrane remodeling by targeting RTN4 induces pyroptosis to facilitate antitumor immune

Mei-Mei Zhao[1,#], Ting-Ting Ren[2,#], Jing-Kang Wang[1], Lu Yao[1], Ting-Ting Liu[1], Ji-Chao Zhang[1], Yang Liu[3], Lan Yuan[4], Dan Liu[4], Jiu-Hui Xu[2], Peng-Fei Tu[1], Xiao-Dong Tang[2,*], Ke-Wu Zeng[1,*]

1. State Key Laboratory of Natural and Biomimetic Drugs, School of Pharmaceutical Sciences, Peking University, Beijing 100191, China
2. Beijing Key Laboratory of Musculoskeletal Tumor, Peking University People's Hospital, Beijing 100044, China
3. Center of Basic Medical Research, Institute of Medical Innovation and Research, Peking University Third Hospital, Beijing 100191, China
4. Proteomics Laboratory, Medical and Healthy Analytical Center, Peking University Health Science Center, Beijing 100191, China.

[#]These authors contributed equally to this work.
*Correspondence: tangxiaodong@pkuph.edu.cn (XD Tang), ZKW@bjmu.edu.cn (KW Zeng)

Supplemental Figures

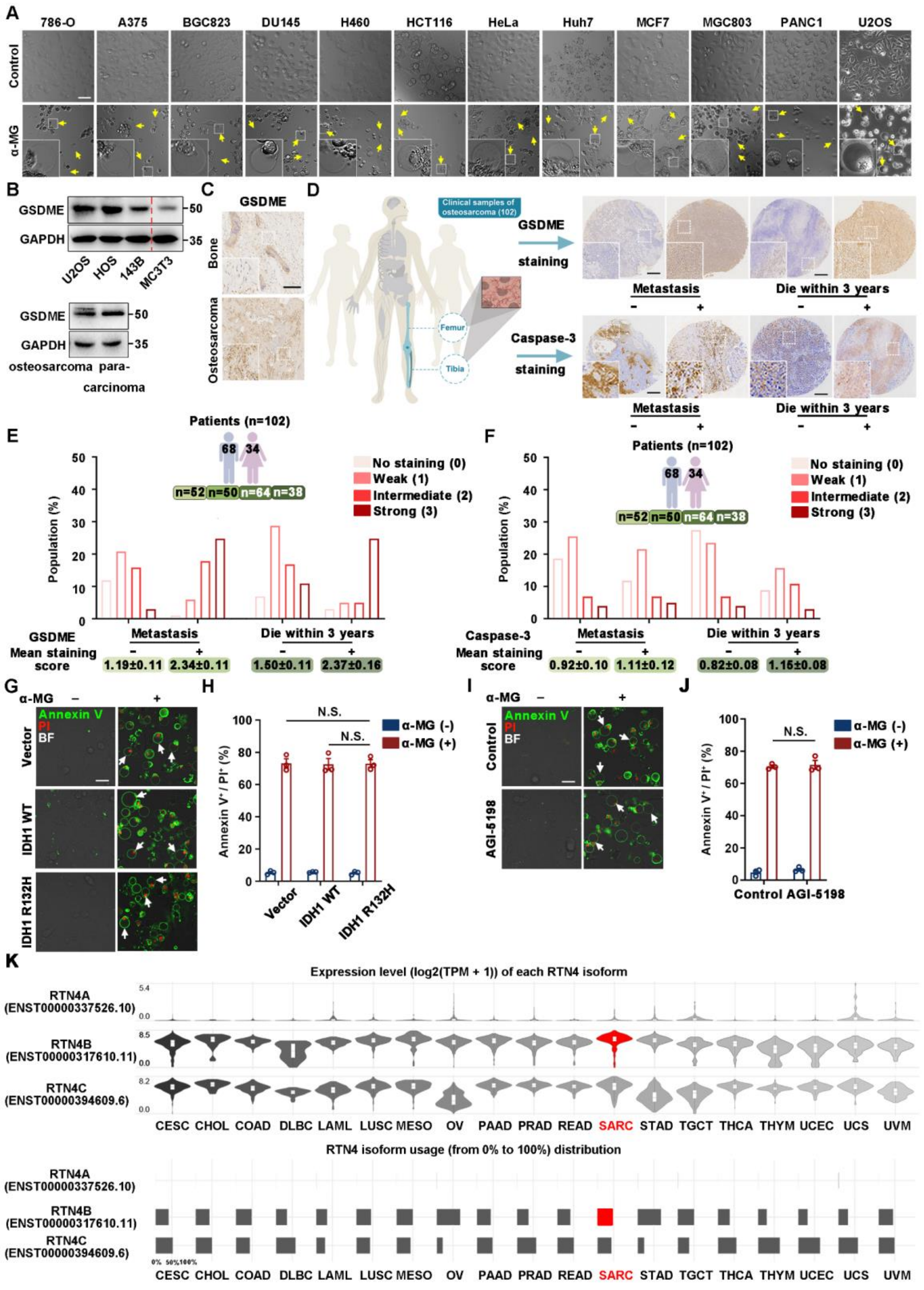

Figure S1. Discovery of RTN4 as a functional protein in pyroptosis

(A) α-MG induced pyroptosis in various cancer cells by morphological analysis (scale bar: 25 μm). **(B)** GSDME expressions in different osteosarcoma cells (U2OS, HOS, 143B) or tissues are higher than the control MC3T3 cells or para-carcinoma tissues by

western blot. **(C)** GSDME expression in osteosarcoma tissues was higher than that in normal bone tissues (Scale bar: 50 μm). **(D)** 3 paraffin-embedded chips of osteosarcoma patients were used for GSDME and caspase-3 immunohistochemistry. **(E, F)** Assessment of GSDME and caspase 3 expression via immunohistochemical staining scoring: correlation with tumor metastasis and 3-year patient mortality. **(G, H)** IDH1-R132H mutation had no impact on α-MG-induced pyroptosis as assessed by Annexin V-PI double staining (scale bar: 25 μm). **(I, J)** IDH1-R132H inhibitor AGI-5198 did not impact the pyroptotic phenotype induced by α-MG in U2OS cells as assessed by Annexin V-PI double staining (scale bar: 25 μm). N.S.: no significance. **(K)** The violin-plots showed the expression level ($\log_2$(TPM + 1)) of each isoform in RTN4 while the bar-plot panel presented the isoform usage (from 0% to 100%) distribution. SARC: Sarcoma.

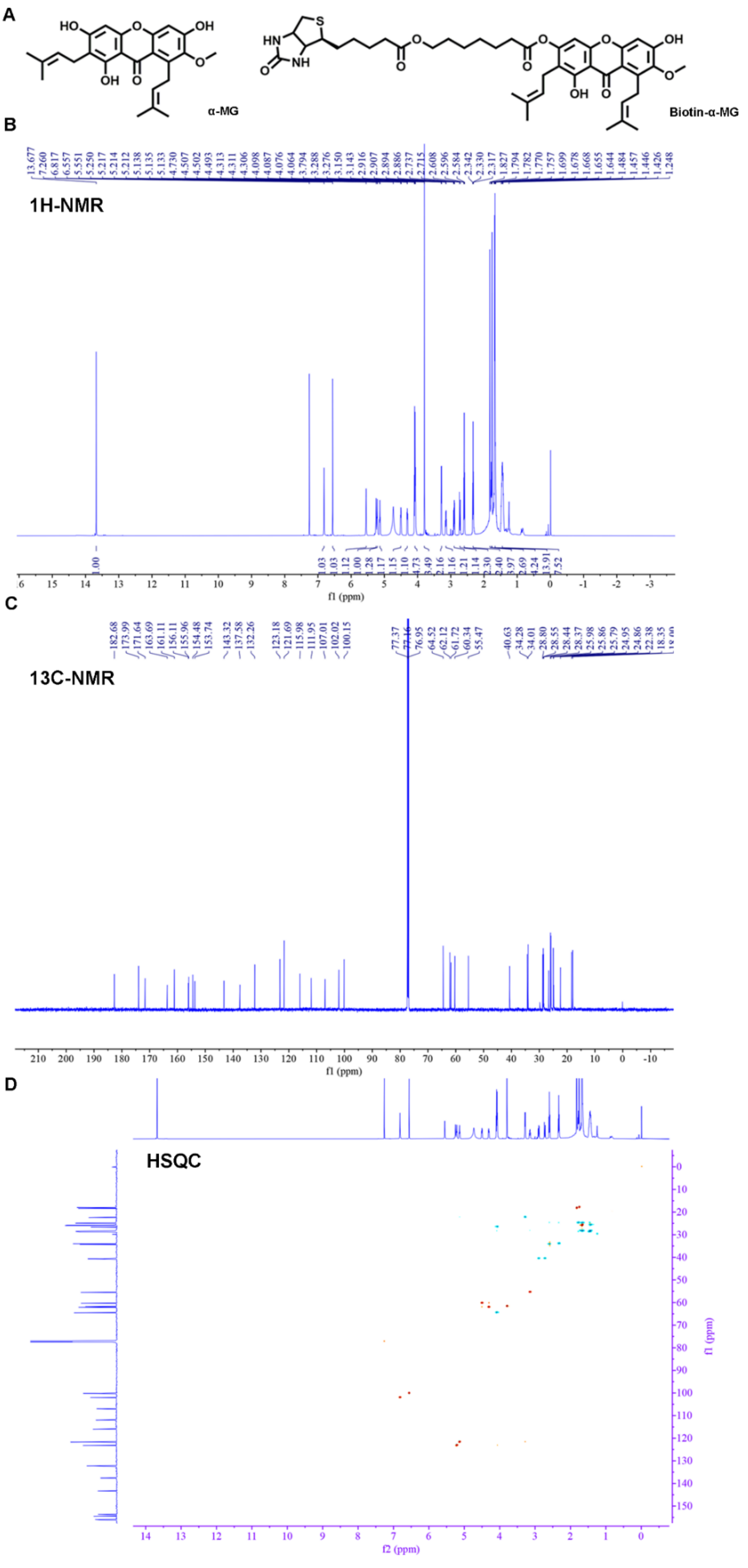
A
α-MG
Biotin-α-MG
B
1H-NMR
f1 (ppm)
C
13C-NMR
f1 (ppm)
D
HSQC
f1 (ppm)
f2 (ppm)

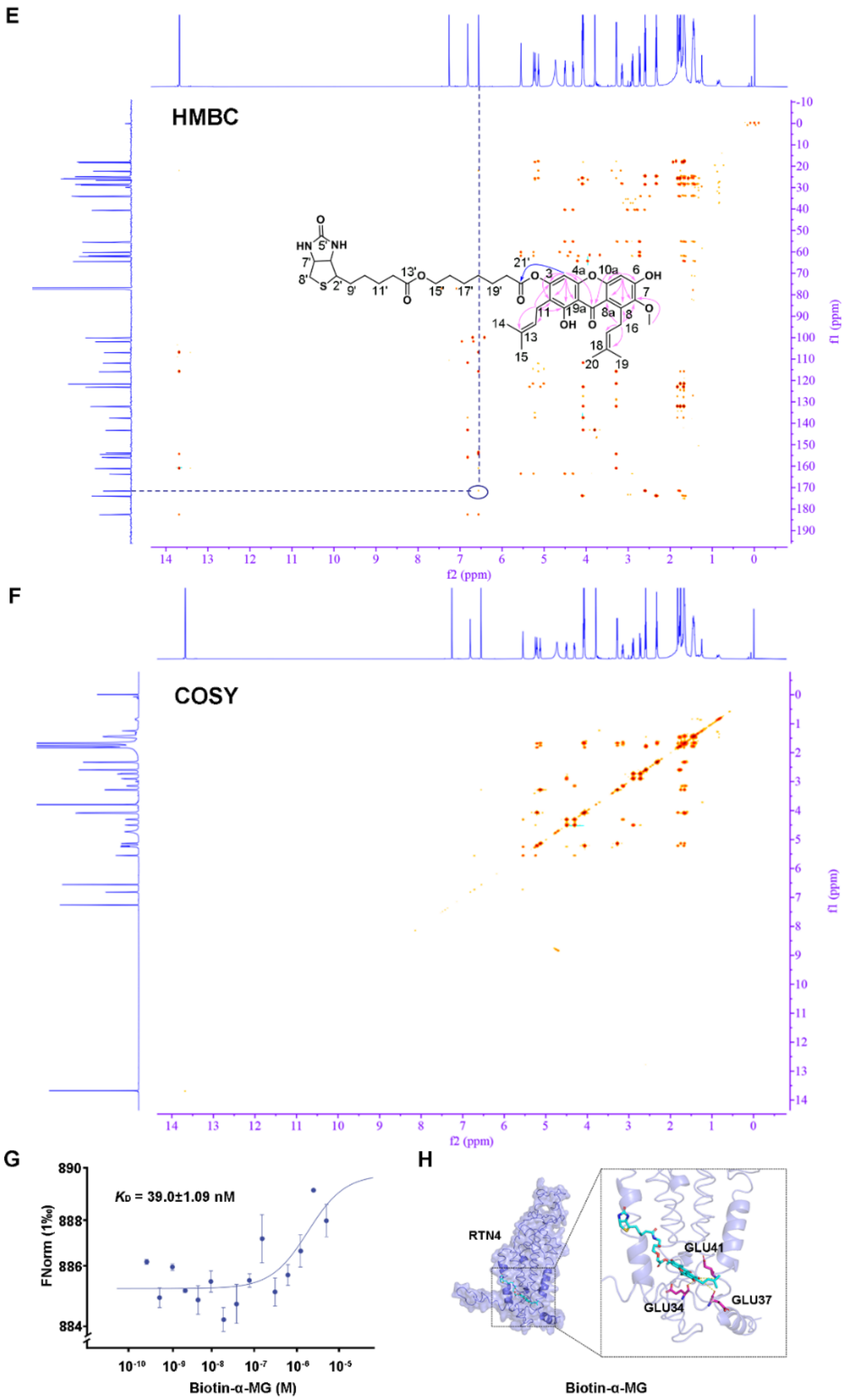

Figure S2. Spectrometric identification of biotin-α-MG probe

(A) Chemical structures of α-MG and biotin-α-MG. **(B)** 1H-NMR (CDCl$_3$, 600 MHz) of biotin-α-MG: δH 6.56 (1H, s, H-4), 6.82 (1H, s, H-5), 3.79 (1H, s, 7-OCH$_3$), 3.28 (1H, d, J = 7.2 Hz, H-11), 5.14 (1H, d, J = 7.2 Hz, H-12), 1.76 (3H, s, H-14), 1.67 (3H,

s, H-15), 4.08 (2H, overlapped, H-16), 5.21 (1H, d, J = 7.2 Hz, H-17), 1.84 (3H, s, H-19), 1.68 (3H, s, H-20), 3.15 (1H, m, H-2'), 4.31 (1H, dd, J = 7.2, 4.8 Hz, H-3'), 5.55 (1H, s, H-4'), 5.25 (1H, s, H-6'), 4.50 (1H, dd, J = 7.2, 4.8 Hz, H-7'), 2.72 (1H, d, J = 13.2 Hz, H-8'a), 2.90 (1H, 1H, dd, J =13.2, 4.8 Hz, H-8'b), 1.67 (2H, overlapped, H-9'), 1.42–1.48 (10H, overlapped, H-10'–11', 16'–18'), 2.33 (2H, t, J = 7.2 Hz, H-12'), 4.10 (2H, overlapped, H-15'), 1.79 (2H, m, H-19'), 2.60 (2H, t, J = 7.2 Hz, H-20'). **(C)** 13C NMR ($CDCl_3$, 125 MHz) of biotin-α-MG: δC 161.1 (C-1), 116.0 (C-2), 154.5 (C-3), 100.1 (C-4), 153.7 (C-4a), 102.0 (C-5), 156.0 (C-6), 143.3 (C-7), 61.7 (7-OCH_3), 137.6 (C-8), 111.9 (C-8a), 182.7 (C-9), 107.0 (C-9a), 156.0 (C-10a), 22.4 (C-11), 121.7 (C-12), 132.3 (C-13), 18.0 (C-14), 25.9 (C-15), 26.6 (C-16), 123.2 (C-17), 132.2 (C-18), 18.4 (C-19), 26.0 (C-20), 55.5 (C-2'), 62.1 (C-3'), 163.7 (C-5'), 60.3 (C-7'), 40.6 (C-8'), 25.0 (C-9'), 28.6 (C-10'), 28.8 (C-11'), 34.0 (C-12'), 174.0 (C-13'), 64.5 (C-15'), 28.4 (C-16'), 25.8 (C-17'), 28.4 (C-18'), 24.9 (C-19'), 34.3 (C-20'), 171.6 (C-21'). **(D)** HSQC, **(E)** HMBC and **(F)** ^1H–^1H COSY spectroscopy of biotin-α-MG. **(G)** The binding of biotin-α-MG to RTN4 was determined by microscale thermophoresis (MST). **(H)** Molecular docking of Biotin-α-MG and RTN4.

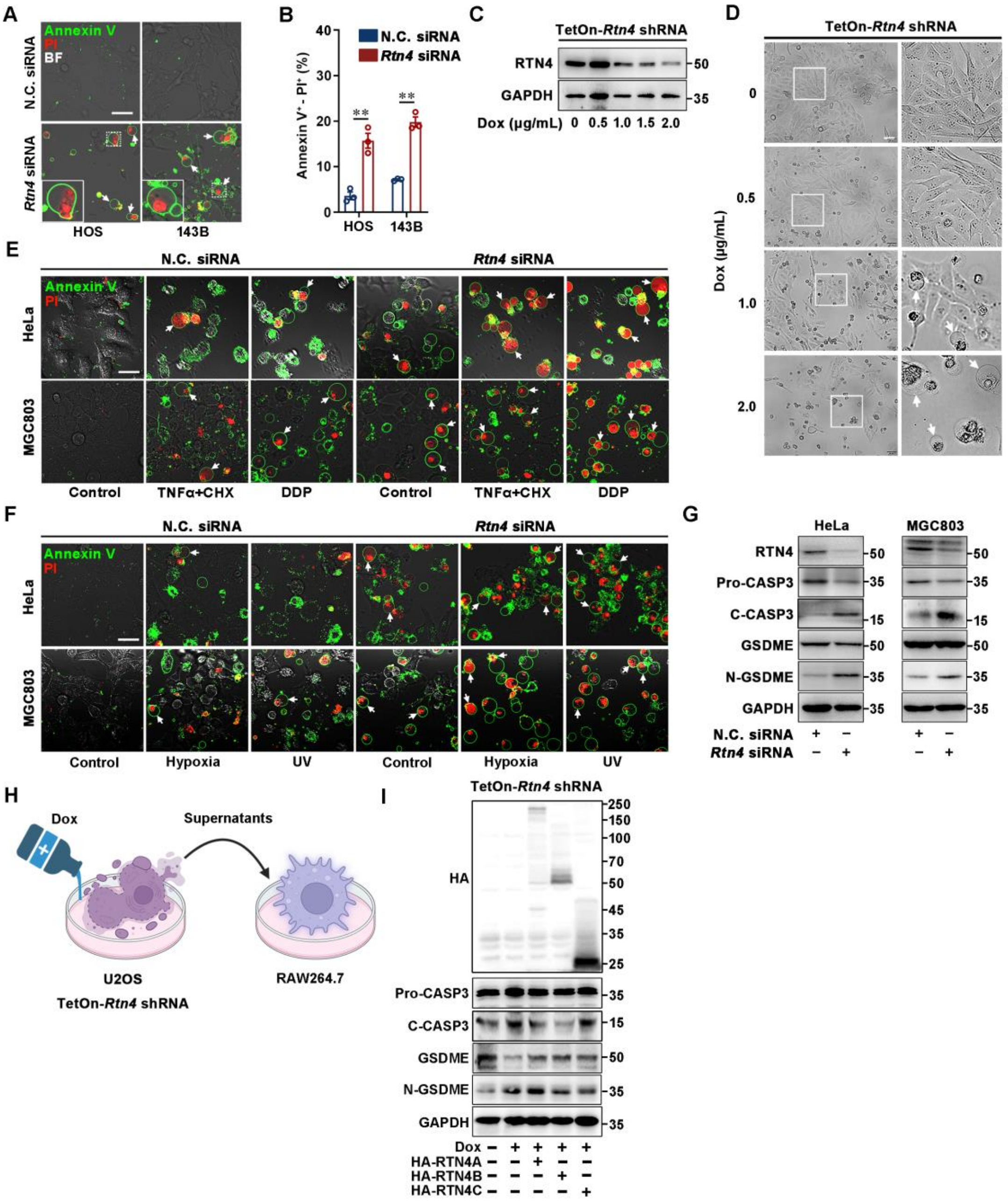

Figure S3. RTN4 deficiency significantly promotes pyroptosis phenotype

(A) RTN4 siRNA induced obvious pyroptosis morphology in osteosarcoma cell lines HOS and 143B by Annexin V-PI double staining (scale bar: 25 μm). **(B)** RTN4 siRNA increased the ratio of pyroptotic cells by Annexin V-PI flow analysis. **(C)** Dox down-regulated the expression of RTN4 in U2OS cells carrying TetOn-RTN4 shRNA in a concentration-dependent manner by immunoblot assay. **(D)** Phenotype-based concentration screen of TetOn-RTN4 shRNA system in U2OS cells (scale bar: 25 μm). **(E)** RTN4 siRNA enhanced the pyroptosis phenotype in TNFα/CHX or DDP-treated HeLa and MGC803 cells by Annexin V-PI double staining. **(F)** Hypoxia and UV radiation showed synergetic effects with RTN4 siRNA on pyroptosis induction by

Annexin V-PI double staining in HeLa and MGC803 cells (scale bar: 25 μm). **(G)** RTN4 siRNA activated caspase-3/GSDME signal pathway by immunoblot assay. **(H)** Schematic diagram of cell communication between U2OS cells with RAW264.7 macrophages for immune activation. **(I)** Overexpression of RTN4B isoform but not RTN4A or RTN4C rescued caspase 3 and GSDME cleavage in TetOn-RTN4 shRNA U2OS cell lines.

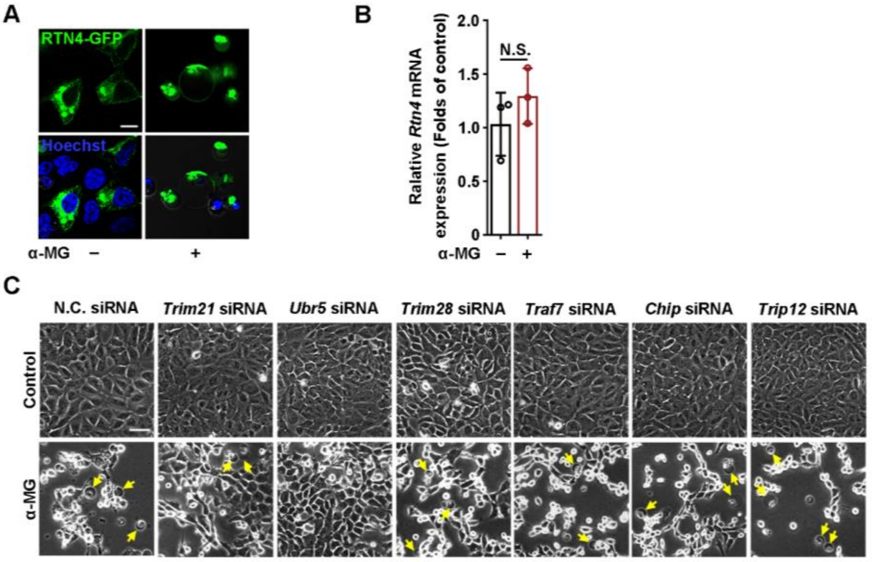

Figure S4. α-MG serves as a RTN4 degrader by recruiting E3 ligase UBR5 in ubiquitin-proteasome system

(A) α-MG reduced RTN4-GFP expression by fluorescence imaging in U2OS cells with RTN4-GFP overexpression (scale bar: 10 μm). **(B)** α-MG showed no significant effect on RTN4 mRNA expression by qPCR assay. N.S.: no significance vs. control group. **(C)** UBR5 knockdown but not TRIM21, TRIM28, TRAF7, CHIP or TRIP12 effectively inhibited α-MG-induced pyroptotic bodies formation (scale bar: 25 μm).

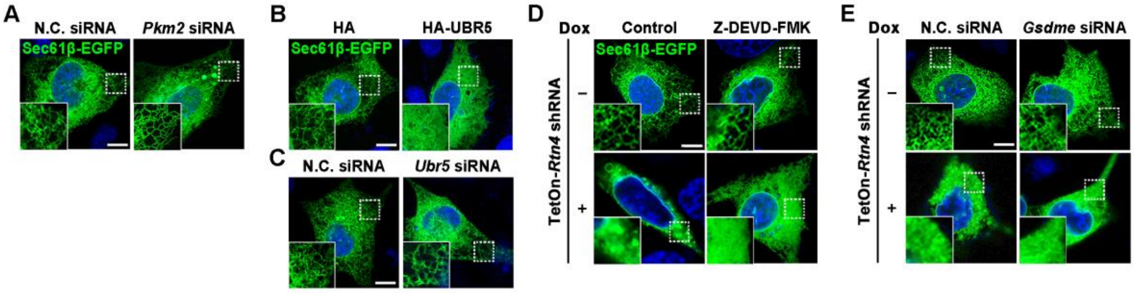

Figure S5. The influence of Caspase-3/GSDME/UBR5/PKM2 on ER morphology in U2OS cells expressing Sec61β-EGFP by Airyscan super-resolution microscopy.

(A) PKM2 siRNA failed to induce ER tubules-to-sheets transition. **(B, C)** UBR5 overexpression or knockdown had no obvious effect on ER membrane curvature alteration. **(D)** Caspase-3 inhibitor Z-DEVD-FMK had no effects on RTN4-mediated ER membrane curvature remodeling. **(E)** GSDME siRNA had no effects on RTN4-mediated ER membrane curvature remodeling (scale bar: 10 μm).

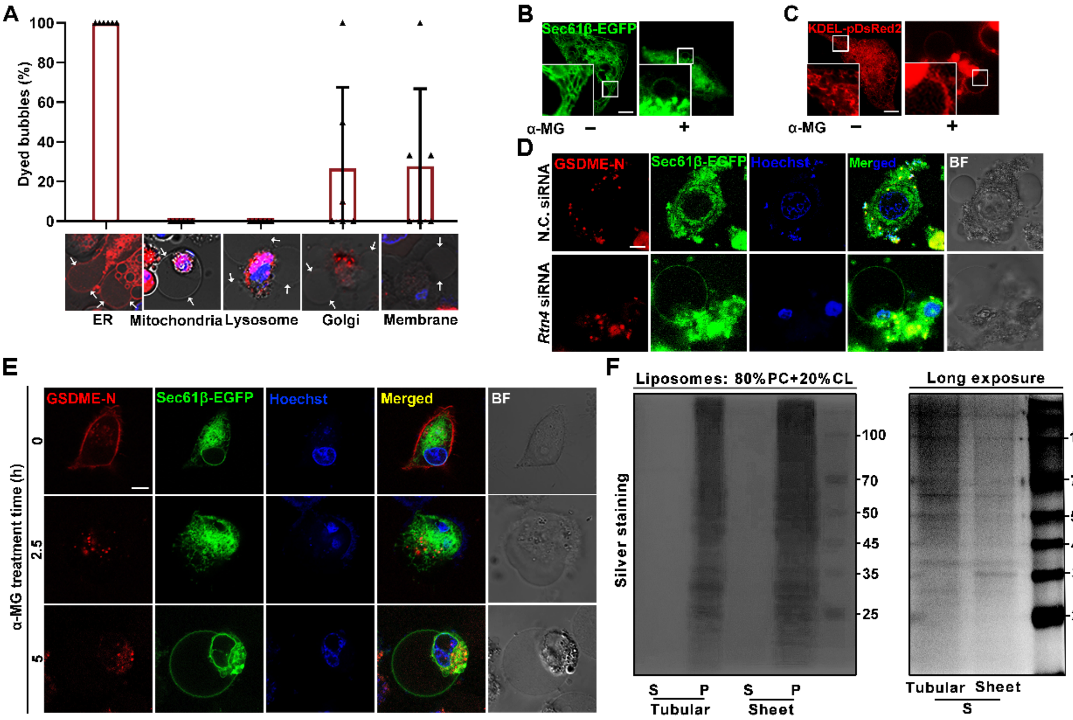

Figure S6. RTN4 knockdown promotes ER-membrane fusion to the 'bubble' structures.

(A) Fluorescence probes (ER tracker, Mito tracker, Lyso tracker, Golgi tracker and Cell membrane tracker DiI) tracking of the 'bubble' structures induced by α-MG. **(B)** Sec61β-EGFP-labelled ER membrane or **(C)** pDsRed2-KDEL-labelled ER lumen was highly expressed on α-MG-induced 'bubble' structures (scale bar: 10 μm). **(D)** RTN4 knockdown prompted Sec61β-EGFP-labelled ER membrane translocation from GSDME-N pores to the 'bubble' structures. **(E)** α-MG induced Sec61β-labelled ER membrane translocation from GSDME-N pores to the 'bubble' structures. **(F)** Sheet ER proteins were more likely to bind with liposomes rather than tubular ER proteins as shown by sliver staining. S: liposome-free supernatants; P: liposome pellets.

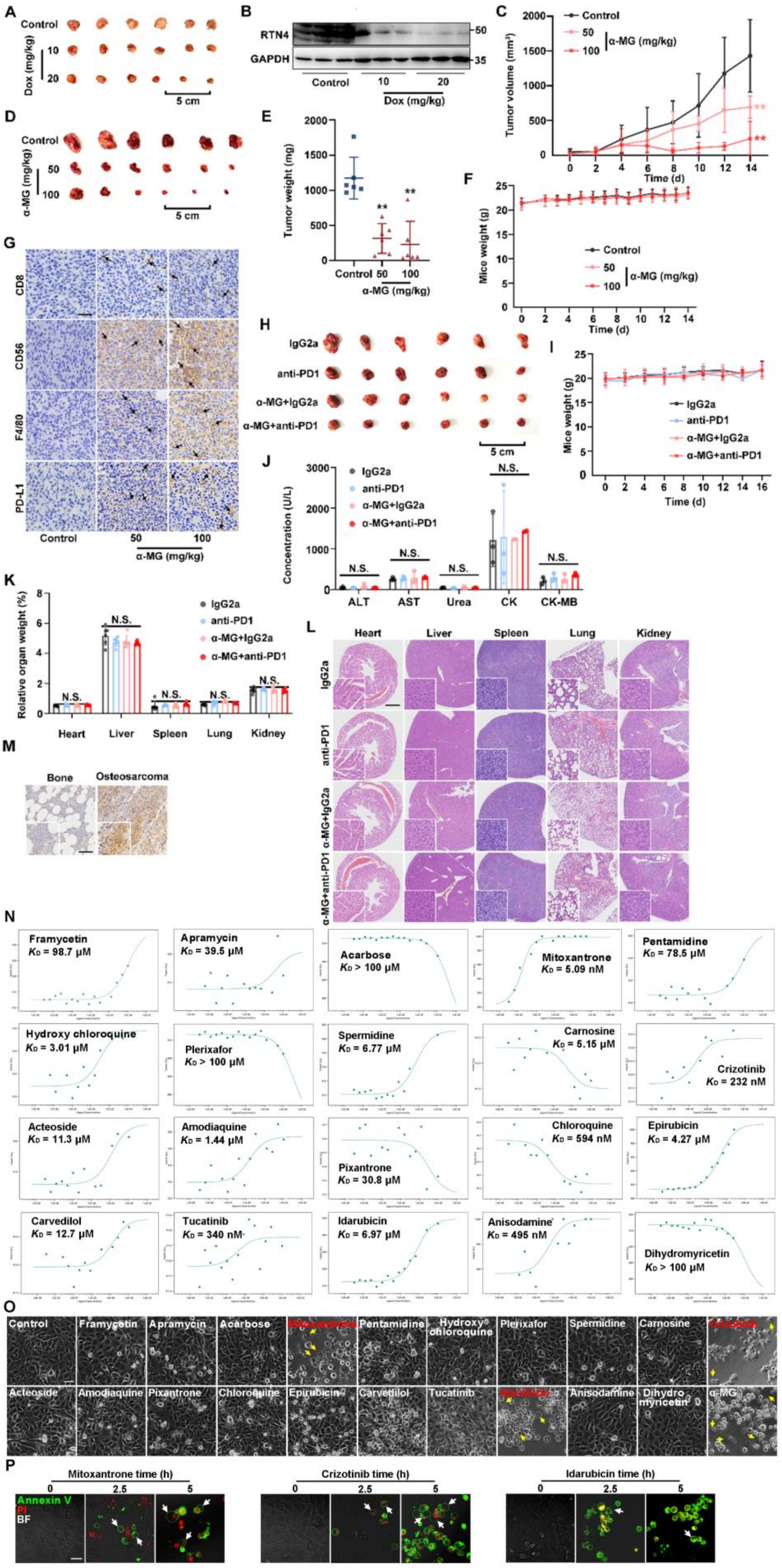
A
Control
10
20
Dox (mg/kg)
5 cm
B
RTN4
GAPDH
50
35
Control
10
20
Dox (mg/kg)
C
Control
50
100
α-MG (mg/kg)
Tumor volume (mm³)
Time (d)
D
Control
50
100
α-MG (mg/kg)
5 cm
E
Tumor weight (mg)
Control
50
100
α-MG (mg/kg)
F
Mice weight (g)
Control
50
100
α-MG (mg/kg)
Time (d)
G
CD8
CD56
F4/80
PD-L1
Control
50
100
α-MG (mg/kg)
H
IgG2a
anti-PD1
α-MG+IgG2a
α-MG+anti-PD1
5 cm
I
Mice weight (g)
IgG2a
anti-PD1
α-MG+IgG2a
α-MG+anti-PD1
Time (d)
J
Concentration (U/L)
IgG2a
anti-PD1
α-MG+IgG2a
α-MG+anti-PD1
N.S.
ALT
AST
Urea
CK
CK-MB
K
Relative organ weight (%)
IgG2a
anti-PD1
α-MG+IgG2a
α-MG+anti-PD1
N.S.
Heart
Liver
Spleen
Lung
Kidney
L
Heart
Liver
Spleen
Lung
Kidney
IgG2a
anti-PD1
α-MG+IgG2a
α-MG+anti-PD1
M
Bone
Osteosarcoma
N
Framycetin KD = 98.7 μM
Apramycin KD = 39.5 μM
Acarbose KD > 100 μM
Mitoxantrone KD = 5.09 nM
Pentamidine KD = 78.5 μM
Hydroxy chloroquine KD = 3.01 μM
Plerixafor KD > 100 μM
Spermidine KD = 6.77 μM
Carnosine KD = 5.15 μM
Crizotinib KD = 232 nM
Acteoside KD = 11.3 μM
Amodiaquine KD = 1.44 μM
Pixantrone KD = 30.8 μM
Chloroquine KD = 594 nM
Epirubicin KD = 4.27 μM
Carvedilol KD = 12.7 μM
Tucatinib KD = 340 nM
Idarubicin KD = 6.97 μM
Anisodamine KD = 495 nM
Dihydromyricetin KD > 100 μM
O
Control
Framycetin
Apramycin
Acarbose
Mitoxantrone
Pentamidine
Hydroxy chloroquine
Plerixafor
Spermidine
Carnosine
Crizotinib
Acteoside
Amodiaquine
Pixantrone
Chloroquine
Epirubicin
Carvedilol
Tucatinib
Idarubicin
Anisodamine
Dihydro myricetin
α-MG
P
Mitoxantrone time (h)
Crizotinib time (h)
Idarubicin time (h)
0
2.5
5
Annexin V
PI
BF

Figure S7. Translational study of targeting RTN4 for anticancer therapy

(A) Intratumor injection of Dox (10 and 20 mg/kg) reduced the tumor sizes. **(B)** Dox treatment reduced RTN4 expression. **(C-E)** Oral administration of RTN4 degrader α-MG (50 and 100 mg/kg) retarded the tumor growth, down-regulated the tumor sizes and decreased the tumor weights. **(F)** Oral administration of α-MG (50 and 100 mg/kg) had no effect on body weights. **(G)** α-MG treatment activated antitumor immunity by immunohistochemical analysis. Scale bar: 100 μm. **(H)** α-MG administration coupled with anti-PD1 treatment displayed a marked synergistic action in reducing tumor sizes. **(I)** α-MG administration coupled with anti-PD1 treatment displayed no obvious adverse effects on body weights. **(J)** Co-treatment of α-MG with anti-PD1 antibodies showed no effects on serum biochemical indicators. **(K)** Co-treatment of α-MG with anti-PD1 antibodies did not change relative organ weights. **(L)** Co-treatment of α-MG with anti-PD1 antibodies did not change the histomorphology of heart, liver, spleen, lung, and kidney by immunohistochemical staining (Scale bar: 50 μm). **(M)** RTN4 expression in osteosarcoma tissues was higher than that in normal bone tissues (Scale bar: 50 μm). Data were presented as mean ± SD (n = 6). $^{**}P < 0.01$ vs. control group. N.S.: no significance vs. control group. **(N)** MST assay showed that a majority of candidates exhibited a strong binding affinity to RTN4. **(O)** Characteristic analysis of pyroptosis in U2OS cells under bright-field microscopy. Each compound was administered with 80 μM for 5 h (scale bar: 25 μm). **(P)** Mitoxantrone, Crizotinib and Idarubicin time-dependently increased the proportion of pyroptosis by Annexin V-PI double staining (scale bar: 25 μm).

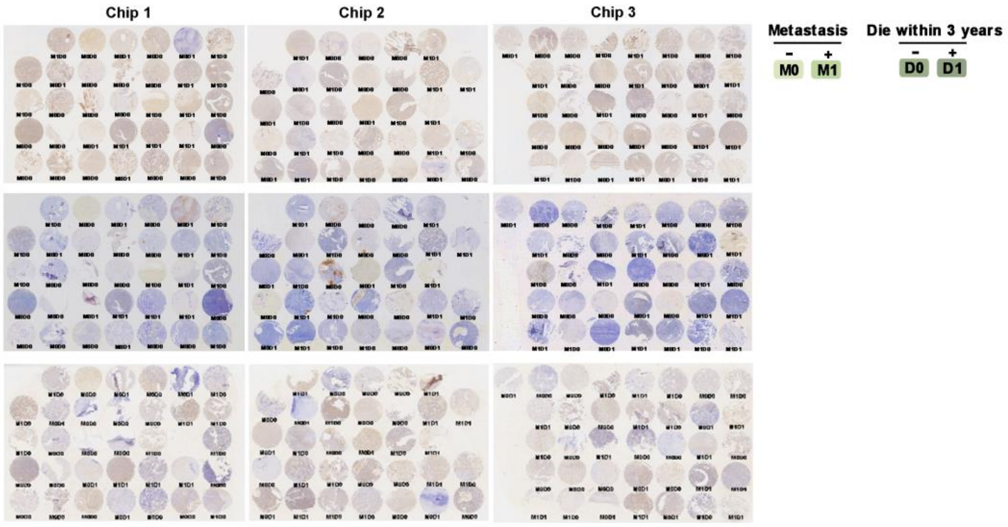

Figure S8. Paraffin-embedded chips from osteosarcoma patients were used for GSDME/caspase-3/RTN4 immunohistochemistry. M0: tumor without metastasis; M1: tumor metastasized: D0: patient survived within 3 years; D1: patients died within

3 years.

Supplemental Tables

Supplementary Table 1. siRNA sequences for transfection

RTN4	5’-3’ (sense)	GGCACAGAUAGAUCAUUAUTT
siRNA	5’-3’ (antisense)	AUAAUGAUCUAUCUGUGCCTT
GSDME	5’-3’ (sense)	GCAUGAUGAAUGACCUGACUUTT
siRNA	5’-3’ (antisense)	AAGUCAGGUCAUUCAUCAUGCTT
PKM2	5’-3’ (sense)	CCAUAAUCGUCCUCACCAATT
siRNA	5’-3’ (antisense)	UUGGUGAGGACGAUUAUGGTT
UBR5	5’-3’ (sense)	CAACUUAGAUCUCCUGAAA
siRNA	5’-3’ (antisense)	GAUUGUAGGUUACUUAGAA
Negative	5’-3’ (sense)	UUCUCCGAACGUGUCACGUTT
siRNA	5’-3’ (antisense)	ACGUGACACGUUCGGAGAATT

Supplementary Table 2. Primer pairs for real-time PCR

Gene	Sequence
Tnf-α	F: 5’-AAGCAAGCAGCCAACCAG-3’
	R: 5’-CCACAAGCAGGAATGAGAAGA-3’
Il-6	F: 5’-ACAAAGCCAGAGTCCTTCAGAGAGA-3’
	R: 5’-ACAAAGCCAGAGTCCTTCAGAGAGA-3’
iNos	F: 5’-ACCCCTGTGTTCCACCAGGAGATGTTGAA-3’
	R: 5’-TGAAGCCATGACCTTTCGCATTAGCATGG-3’
Il-1β	F: 5’-TGGAGAAGCTGTGGCAGCTACCT-3’
	R: 5’-GAACGTCACACACCAGCAGGTT-3’
Atlastin-1	F: 5’-CAGCACCTCCAGCTTTTCACTG-3’
	R: 5’-CACCACCATCGGCTCCATATGA-3’
Rtn4	F: 5’-CAGCACCTCCAGCTTTTCACTG-3’
	R: 5’-ACTGTCAATGAAAGCAGCAGGA-3’
β-actin	F: 5’-TTTTGGCTATACCCTACTGGCA-3’
	R: 5’-CTGCACAGTCGTCAGCATATC-3’
